# Impact of energy turnover on the regulation of glucose homeostasis in healthy subjects

**DOI:** 10.1038/s41387-019-0089-6

**Published:** 2019-08-08

**Authors:** Franziska Büsing, Franziska Anna Hägele, Alessa Nas, Mario Hasler, Manfred James Müller, Anja Bosy-Westphal

**Affiliations:** 1 Institute of Human Nutrition and Food Science, Christian-Albrechts University of Kiel, Kiel, Germany; 2 Institute of Nutritional Medicine, University of Hohenheim, Stuttgart, Germany; 3 Applied Statistics, Faculty of Agricultural and Nutritional Sciences, Christian-Albrechts University of Kiel, Kiel, Germany

**Keywords:** Randomized controlled trials, Risk factors

## Abstract

**Objective:**

Sedentary lifestyle increases the risk of type 2 diabetes. The aim of this study was to investigate the impact of different levels of energy turnover (ET; low, medium, and high level of physical activity and the corresponding energy intake) on glucose metabolism at zero energy balance, caloric restriction, and overfeeding.

**Methods:**

Sixteen healthy individuals (13 men, 3 women, 25.1 ± 3.9 years, BMI 24.0 ± 3.2 kg/m^2^) participated in a randomized crossover intervention under metabolic ward conditions. Subjects passed 3 × 3 intervention days. Three levels of physical activity (PAL: low 1.3, medium 1.6, and high 1.8 achieved by walking at 4 km/h for 0, 3 × 55, or 3 × 110 min) were compared under three levels of energy balance (zero energy balance (EB): 100% of energy requirement (Ereq); caloric restriction (CR): 75% Ereq, and overfeeding (OF): 125% Ereq). Continuous interstitial glucose monitoring, C-peptide excretion, and HOMA–IR, as well as postprandial glucose and insulin were measured.

**Results:**

Daylong glycemia and insulin secretion did not increase with higher ET at all conditions of energy balance (EB, CR, and OF), despite a correspondingly higher CHO intake (Δ low vs. high ET: +86 to 135 g of CHO/d). At CR, daylong glycemia (*p* = 0.02) and insulin secretion (*p* = 0.04) were even reduced with high compared with low ET. HOMA–IR was impaired with OF and improved with CR, whereas ET had no effect on fasting insulin sensitivity. A higher ET led to lower postprandial glucose and insulin levels under conditions of CR and OF.

**Conclusion:**

Low-intensity physical activity can significantly improve postprandial glycemic response of healthy individuals, independent of energy balance.

## Introduction

Higher postprandial glycemia even below the diabetic threshold has been shown to be a risk factor for cardiovascular disease^1,2^. In addition, a higher glycemic load was positively associated with the risk of type 2 diabetes in a meta-analysis of prospective cohort studies^3^. Exercise could compensate the negative effects of a high glycemic load (GL) western diet, because it mitigates postprandial glycemia by non-insulin-mediated glucose uptake (NIMGU)^4–6^. Numerous studies with high-intensity exercise showed lower postprandial glucose levels or/and an improved insulin sensitivity in healthy participants^7^, as well as in patients with diabetes^8^. However, recommendation of high-intensity exercise for primary prevention has limitations with respect to injury in high-risk groups like untrained obese and elderly subjects. Nygaard et al. investigated the effect of low-intensity physical activity on regulation of glycemia in healthy subjects. The authors found a 31.2% decrease of postprandial glucose by a slow 40-min postmeal walk compared with control in healthy women aged >50 years^9^. Manohar et al. confirm this result in 12 healthy control subjects and found a significant reduction of the postprandial glucose AUC of 53.1% by walking at a speed of 1.9 km/h^10^.

The timing of physical activity may also impact postprandial glycemia, because of diurnal variations in energy metabolism and insulin secretion^11^. In patients with type 2 diabetes, most^12–16^ but not all^17^ studies showed that exercise or moderate walking after meals had a greater benefit on postprandial glycemia compared with premeal exercise, whereas premeal exercise had a greater impact on improvement of fat oxidation^18^.

A major drawback of all previous studies is the condition of uncontrolled energy intake with physical activity. Because physical activity leads to an increase in energy expenditure and increases energy intake, only partially to the corresponding enhanced energy requirement (Ereq)^19^, a negative energy balance (EB) is likely created that impacts glucose metabolism. Zero EB, where energy intake corresponds to energy expenditure, is therefore necessary to investigate the effect of physical activity on regulation of glycemia without the confounder of negative EB. A condition of fixed EB at a varying physical activity level is defined as energy turnover (ET) or energy flux. A low ET, i.e., a low energy intake with a low energy expenditure, resembles an inactive lifestyle, whereas a high ET can be achieved by an increase in physical activity at a correspondingly higher energy intake. A high ETmight compensate for the negative impact of short-term overfeeding (OF) on glucose metabolism. Sedentary behavior is known to facilitate a positive EBe, e.g., over the weekend, on holidays, at periods of celebration, or during vacations^20,21^. There is evidence that one day of sitting without adjusting for the lower energy expenditure already leads to a reduced insulin action (−39%) similar to the changes reported after longer periods of bed rest or a large reduction in ambulation^22^.

The aim of the study was to investigate the impact of low, medium, and high ET obtained by different durations of low-intensity physical activity (performed after the meals) on the regulation of basal and circadian glucose metabolism at zero EB. The impact of these three levels of ET on glucose metabolism was also examined under randomized conditions of controlled OF and caloric restriction (CR).

## Methods

Sixteen healthy subjects were recruited via announcements on social networks and at the campuses of the University of Stuttgart and Hohenheim. Inclusion criteria were age between 20 and 40 years and a normal physical activity in daily routine. Exclusion criteria for enrollment included regular intake of supplements, chronic disease, smoking, claustrophobia, and special diets or any food intolerances. Subjects with chronic diseases or regular intake of medication on a daily basis, except birth control pills (one case) were excluded from participation. The study was carried out at the Institute of Nutritional Medicine, University of Hohenheim, Stuttgart, Germany, from December 2016 to March 2018.

### Study design

**Fig. 1:**
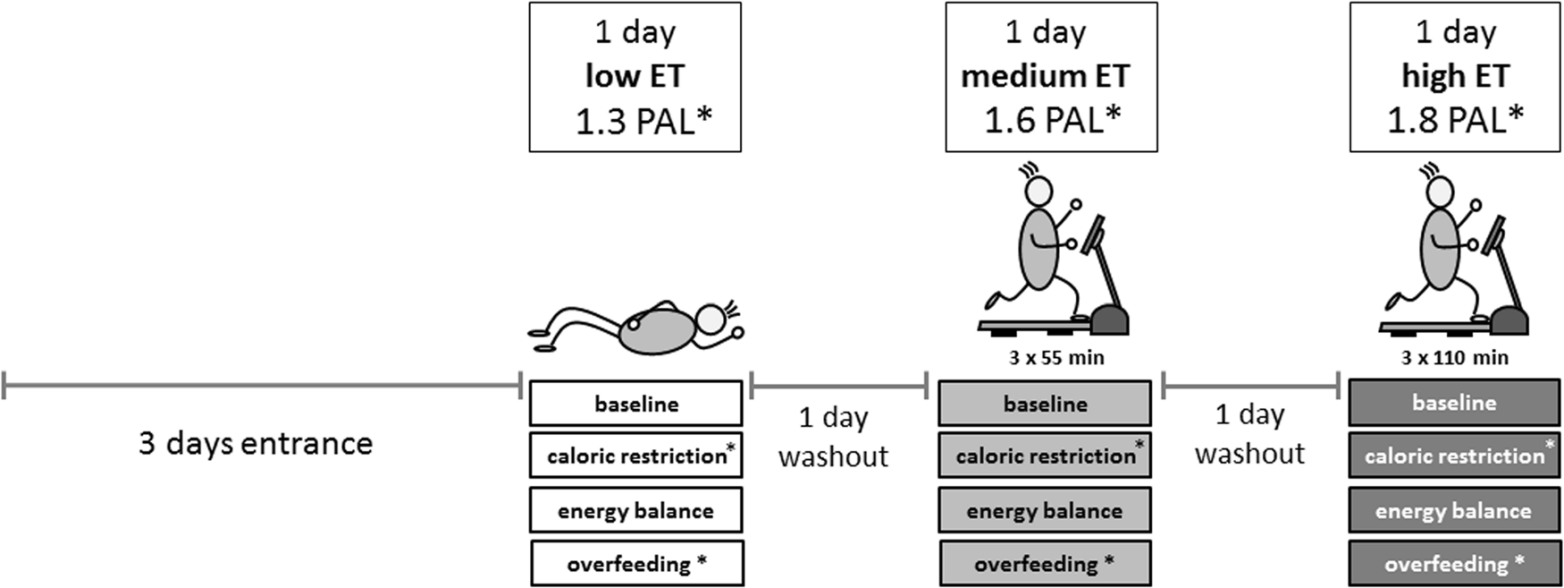
**Schematic study protocol**. *randomized order

The randomized controlled crossover intervention comprised three 1-week interventions: zero EB, CR −25 Ereq%, and OF +25 Ereq%. Each week was performed under three different levels of ET: one inactive day (PAL 1.3 = low ET), one day of normal physical activity (PAL 1.6 = medium ET) and one day of high physical activity (PAL 1.8 = high ET; Fig. 1). The three intervention days per week were separated by a washout day. A subject who completed the entire study thus went through nine different intervention days and additional 3 days, where the Ereq for low, medium, and high ET was measured with *ad libitum* energy intake (baseline week). Each of the nine intervention days consisted of a 36-h stay in the metabolic chamber. Participants entered the metabolic chamber in the evening before each test day. The measurement period started at 6 a.m. in the morning and ended 24 h later. The sleeping period was constant and always from 10:30 p.m. to 6 a.m. Throughout the whole study period, participants wore an actiwatch (Actiwatch 2, Koninklijke Philips N.V., Amsterdam, The Netherlands), to monitor sleeping periods (duration, start/end, and sleep quality). EB was conducted as the second intervention week, whereas CR and OF were performed in randomized order as the first or third week of study. All intervention days (low, med, and high) were kept in randomized order, which was obtained by block randomization.

The study was registered at clinicaltrials.gov as NCT03361566 and the study protocol was approved by the ethics committee of the Medical Council of Baden-Württemberg, Germany. All participants provided informed written consent before participation.

### Twenty-four-hour energy expenditure

Resting Ereq of all study participants was measured before the baseline week by a hood calorimetry system. Resting Ereq was multiplied by a physical activity level estimated from prescribed physical activity during the baseline week in the metabolic chamber. In order to provide a sufficient amount of food during baseline week ~20% of extra energy was provided and all leftovers were weight back to assess food consumption. Energy expenditure at different levels of activity (baseline week) was measured in a room calorimeter and was used to determine individual Ereq (for details see ref. ^23^). Total energy expenditure (TEE) was determined at a constant flow rate of 120 l/min fresh air by continuously measuring rates of oxygen and carbon dioxide concentrations using the Promethion integrated whole-room indirect calorimeter system (Sable Systems International, Las Vegas, USA) and the Weir equation^24^. Using energy intake and TEE data, individual EB for different ET levels was calculated as ∆EB [%] = (EI/TEE × 100) − 100. The influence of ET on macronutrient oxidation and macronutrient balances is subject of a separate publication (Nas A et al., submitted 2019).

### Body composition

Height was measured using a stadiometer (seca 274, seca GmbH & Co.KG, Hamburg, Germany). Body weight was measured in the morning before breakfast at the beginning and end of each EB condition using a calibrated electronic scale (seca mBCA 515, seca GmbH & Co.KG), in light clothes, without shoes and after voiding. Before starting the study, body composition of the subjects was analyzed using Air Displacement Plethysmography by the BodPod^TM^ Body Composition System (COSMED USA, Inc., Concord, CA, USA) as described elsewhere^25^.

### Standardization of the diet

To ensure equal baseline conditions, each intervention was preceded by a 3-day entrance phase with controlled macronutrient composition of the diet. On intervention days, a strict daily structure with three meals was achieved. Breakfast was served at 7 a.m., lunch at 1 p.m., and dinner at 7 p.m.

The study diet consisted of a lot of energy dense, low fiber convenient food with a high content in saturated fatty acids (e.g., ready-to-serve pizza) and included sweets. The macronutrient composition of 35% fat, 50% carbohydrates, and 15% protein represents a common diet in Germany and was kept constant throughout the whole study period. A constant macronutrient composition was achieved by weighing each food item and by evaluation of the menus with the software Prodi®6 (Scientific Publishing Company, Stuttgart). Meal composition on intervention and washout days as well as during the entrance phase was kept constant. During the baseline week for assessment of Ereq at different levels of ET, as well as in the entrance phase under free living conditions and during the washout days, participants received all food in abundance and leftovers were weighed back to assess energy and macronutrient intake. On all intervention days during EB, CR, and OF, subjects were instructed to eat their entire meal within 30 min without any leftovers. To ensure a controlled dietary intervention, all food consumed throughout the whole study was provided by the institute. For the duration of the entire study, participants were asked to abstain from consumption of alcohol and any additional food or snacks except mineral water, herbal and fruit tea that were allowed ad libitum. GL of a food was calculated by multiplying GI of each food^26^ by the amount of CHO in grams provided by the food and dividing the total by 100^27^.

### Physical activity

Physical activity was recorded using a triaxial activity monitor, activPAL™ (Paltechnologies Ltd., Glasgow, UK). The device was continuously worn on the middle of the thigh fixed with a waterproof patch. ActivPAL™ Professional v7.2.32 software was used for data analysis.

The subjects had to pass three different levels of physical activity depending on the ET. All physical activity levels were achieved by walking with a constant speed of 4 km/h on a treadmill (Kettler Track 9, Kettler GmbH, Ense-Parsit, Germany) for different durations. On the day with low ET (PAL 1.3), subjects were sedentary by sitting or lying throughout the day. On the medium ET day with normal activity, subjects walked on the treadmill three times a day 10 min after finishing meals (PAL = 1.6) for 55 min. On the high ET day, walking units were increased to 110 min each (PAL = 1.8). Before the start of the study, it was tested which walking time and walking speed were appropriate to reach the given PALs. Subjects were told to refrain from exercise during the entrance phase and on the washout days and only follow their usual everyday activities. Due to technical problems with the device, there is no valid data of physical activity of one subject for the whole intervention and two participants had missing data for one EB condition each.

### Assessment of glucose metabolism

In the morning of the intervention days and the following mornings, fasting blood samples were taken. Fasting glucose concentration was determined via hexokinase method (OSR6121, Beckman Coulter, Brea, CA, USA). Fasting serum insulin (Elecsys® Insulin 06923321990, Roche Cobas e801) and 24-h urinary C-peptide excretion were measured by luminescence immunoassay (Elecsys® C-Peptide 06923330990, Roche Cobas e801). Homeostatic model assessment–insulin resistance (HOMA–IR = fasting glucose (mg/dl) × fasting insulin (μU/ml)/405^28^) was used to calculate fasting insulin sensitivity. Daylong glycemia at different levels of ET, was assessed by continuous interstitial glucose measurement (CGM, Dexcom G4 Platinum, Nintamed, Mainz, Germany) for at least 24 h during all nine intervention days. A small sensor was placed at the back of the upper arm in the subcutaneous tissue. Sensor readings were recorded in intervals of 5 min. The CGM-device was calibrated three times a day before meals against fasting capillary blood samples. Incremental AUC was calculated (iAUC) for 18 h (6:00 a.m.–12:00 p.m.) using trapezoidal rule^29^, whereby iAUC includes only the positive AUCe. Glucose variability was described by mean amplitude of glycemic excursions (MAGE-index) and calculated using a published macro^30^. Daylong insulin secretion was assessed by 24-h urinary C-peptide excretion.

Results of the continuous glucose monitoring were adjusted for the difference between fasting serum glucose and fasting CGM_glucose_ (Glucose_CGM_−Glucose_venous_) to calculate postprandial iAUC of glucose. No differences in the results were observed when using the uncorrected glucose values. Plasma samples for the measurement of postprandial insulin were collected 30, 60, and 120 min after each meal in BD^TM^ P800 tubes (Becton Dickinson Inc., Franklin Lakes, USA) and measured using a Bio-Plex Pro^TM^ human Diabetes 3-Plex Kit (Bio-Rad, Hercules, USA). Data analysis was performed with Bio-Plex Manager^TM^ Software 6.1. iAUC was calculated from postmeal insulin values over 2 h.

One participant was excluded for the analysis of daylong insulin secretion (*n* = 15) and HOMA–IR of two subjects was missing for low ET at the condition of EB (*n* = 14).

### Statistical analyses

Data are expressed as mean ± SD. The statistical software R (2017) was used to evaluate the data. Data evaluation started with the definition of an appropriate statistical mixed model^31,32^. The data were assumed to be normally distributed and to be heteroscedastic with respect to different levels of EB and ET. These assumptions are based on a graphical residual analysis. The statistical model included EB (zero EB, CR, and OF) and ET (low, medium, and high), PrePost (before intervention, after intervention), as well as their interaction terms as fixed factors. The ID and EB, nested in ID, were regarded as random factors. Also, the correlations of values between intervention days as well as the correlations between pre and post measurements were taken into account (auto-correlation). Based on this model, a Pseudo R² was calculated^33^ and an analysis of variance (ANOVA) was conducted, followed by multiple contrast tests (e.g., see refs. ^34,35^) in order to compare the several levels of the influence factors. *p* values < 0.05 were considered as statistically significant. Box-and-whisker plots were used to display the distribution of iAUC_CGM_, C-peptide-excretion and HOMA–IR. Based on results of a previous study of our group^36^, a total sample size of *n* = 8 was required to assess these differences in daylong glycemia at a α-level of 0.05 and a power of 95% (hypothesized effect size = 1.4).

## Results

Three women and thirteen men aged 25.1 ± 3.9 years with a BMI 24.0 ± 3.2 kg/m^2^ and a FMI 5.3 ± 3.2 kg/m² were included in this study. According to WHO criteria, ten subjects were normal weight, five overweight and one obese. Regarding body composition, four subjects had a FMI above the age and sex adjusted 95th percentile^37^.

Mean resting energy expenditure of the study participants at baseline was 1788 ± 216 kcal/d. Body weight remained unchanged during all conditions of EB (ΔCR: 0.0 ± 0.3 kg; ΔEB: −0.1 ± 1.1 kg; ΔOF: 0.3 ± 0.3 kg; all *p* > 0.05). Daily step count and PAL were different by design between the three levels of ET in all conditions of EB (all *p* < 0.05; Table 1) but did not differ at the same ET between the three EB conditions (all *p* > 0.05; except low ET: CR vs. OF, *p* < 0.05). Step count was 482, 17706, and 34587 during low, medium, and high ET interventions (mean of all three EBs). Since step count of low ET without any treadmill activity was 436–545 steps/d, step counts of medium and high ET minus these steps is assumed to be reached during treadmill challenge. EB was 3.4%, 0.0%, and 1.6% during zero EB, −20.5%, −19.5%, and −21.0% during CR, and 27.6%, 25.4%, and 24.2% during OF for low, medium, and high ET, respectively (EB vs. CR vs. OF, all *p* < 0.001).

**Table 1:** Physical activity and daily energy-, macronutrient- and glycemic load intake during caloric restriction, zero energy balance and overfeeding with low, medium and high energy turnover (ET)

	Caloric restriction			Energy balance			Overfeeding		
	low ET	med ET	high ET	low ET	med ET	high ET	low ET	med ET	high ET
Physical acticity									
PAL	1.26 ± 0.06	1.52 ± 0.05	1.74 ± 0.07	1.30 ± 0.03	1.57 ± 0.04	1.75 ± 0.05	1.34 ± 0.05	1.55 ± 0.04	1.71 ± 0.05
Steps [counts/d]	545 ± 902	17 618 ± 557	34 527 ± 1 847	464 ± 912	17 710 ± 219	34 414 ± 1 539	436 ± 847	17 790 ± 382	34 821 ± 1 568
Diet									
Energy intake [kcal/d]	1 792 ± 144	2 156 ± 78	2 494 ± 90	2 390 ± 97	2 863 ± 72	3 325 ± 177	2 986 ± 99	3 570 ± 183	4 146 ± 320
CHO intake [g/d]	219.4 ± 20.8	255.1 ± 37.3	308.2 ± 12.0	292.7 ± 13.9	350.1 ± 6.0	406.5 ± 20.3	366.2 ± 11.8	437.2 ± 22.1	506.7 ± 37.9
Fat intake [g/d]	70.5 ± 4.9	84.7 ± 2.6	97.7 ± 5.0	93.7 ± 3.3	112.8 ± 3.9	131.0 ± 7.5	117.3 ± 3.9	140.8 ± 7.0	163.8 ± 12.5
Protein intake [g/d]	66.1 ± 4.0	79.4 ± 3.3	92.0 ± 5.6	88.4 ± 0.0	106.1 ± 5.6	120.3 ± 9.3	109.8 ± 3.0	131.0 ± 8.6	153.3 ± 13.0
Glycemic load [g/d]	124.0 ± 8.9	149.2 ± 4.7	173.8 ± 7.0	166.0 ± 6.5	197.8 ± 5.7	228.9 ± 14.1	206.4 ± 7.0	247.4 ± 13.0	285.9 ± 24.0

Values are means ± SDs; *n* = 16; linear mixed model with multiple contrast tests, results of physical activity were compared at the same level of ET between all energy balance conditions (all *p* > 0.05) and within all energy balances conditions at three levels of ET (all significantly different at *p* < 0.001); results of dietary intake were compared at the same level of ET between all energy balance conditions (all significantly different at *p* < 0.001) and within all energy balances conditions at three levels of ET (all significantly different at *p* < 0.001)

*ET* energy turnover, *CHO* carbohydrate

As intended by study design, total caloric intake, as well as absolute carbohydrate-, fat-, and protein intake increased within each energy balance with higher ET (all *p* < 0.001) and differed between conditions of EB (all *p* < 0.001, Table 1). Macronutrient composition was 49.4 ± 0.5% CHO, 34.9 ± 0.7% fat, and 14.8 ± 0.1% protein and did not differ throughout the different phases of the study. Total daily GL ranged between 123.8 and 285.2 g/d (Table 1). Daily GL was distributed with 27.4% at breakfast, 28.8% at lunch, and 43.8% at dinner.

### Effect on daylong and basal glucose metabolism

**Fig. 2:**
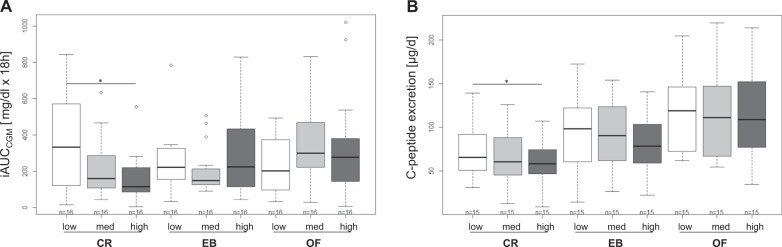
**Comparison between the different levels of ET (low, medium, and high) at different energy balances.** **a** Eighteen-hour iAUCs_CGM_ [mg/dl], *n* = 16; **b** C-peptide excretions [µg/d], *n* = 15; values are means ± SDs; linear mixed model with multiple contrast tests, *p < 0.05 for comparison of ETs; CR caloric restriction, EB energy balance, OF overfeeding, med medium, ET energy turnover

**Fig. 3:**
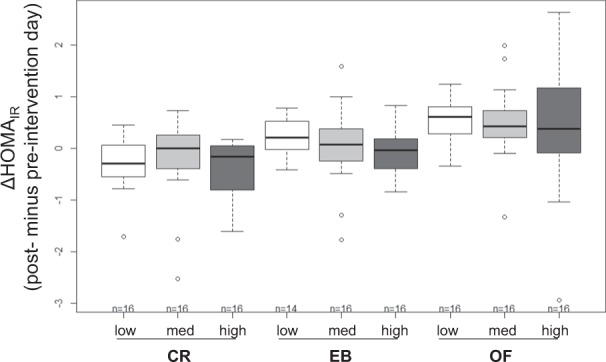
**Comparison of differences in HOMA–IR (post- minus pre-intervention day) between ETs (low, med, and high) within different levels of energy balance (CR, EB, and OF).** Values are means ± SDs; linear mixed model with multiple contrast tests; fasting insulin sensitivity was improved with CR and impaired with OF (all *p* < 0.01); *n* = 14–16; ET energy turnover, med medium, CR caloric restriction, EB energy balance, OF overfeeding

Changes in daylong glycemia and insulin secretion are shown in Fig. 2. During CR, daylong glycemia and insulin secretion were higher with low compared with high ET (*p* < 0.05, Fig. 2). Despite a higher intake of carbohydrates with increasing level of ET (Δ low vs. high ET: +86 to 135 g of CHO/d; Table 1), there was no increase in daylong glycemia and insulin secretion with higher ET during EB and OF. During CR, glucose variability (MAGE-Index) was higher with low ET compared with medium and high ET (low ET: 3.1 ± 1.0, medium ET: 2.3 ± 0.5, high ET: 1.9 ± 0.4; both *p* < 0.05). Fasting insulin sensitivity was improved with CR and impaired with OF (all *p* < 0.01) irrespective of ET (Fig. 3).

### Effect on postprandial glucose metabolism

**Fig. 4:**
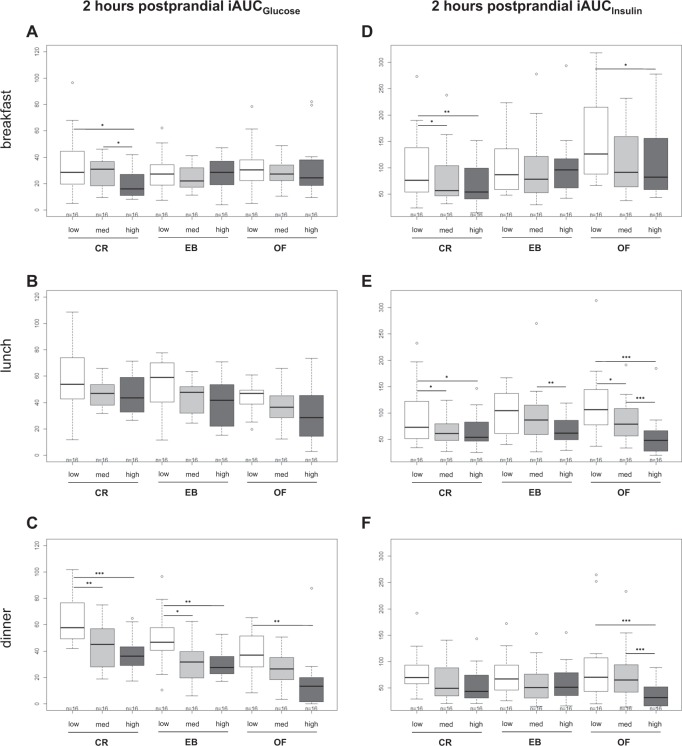
**Comparison of 2** **h postprandial glucose** (**a**–**c**) **and insulin** (**d**–**f**) **iAUCs between the different levels of ET (low, medium, and high) and energy balance (CR, EB, OF) separated by breakfast, lunch, and dinner.** Values are means ± SDs; linear mixed model with multiple contrast tests, *p < 0.05, **p < 0.01, and ***p < 0.001 for comparison of ETs; *n* = 16; ET energy turnover, med medium, CR caloric restriction, EB energy balance, OF overfeeding

Changes in EB did not impact postprandial glucose and insulin responses (EB vs. CR and EB vs. OF; all *p* > 0.05; except postmeal insulin at medium ET CR vs. EB, *p* < 0.05). A higher ET led to lower cumulative postprandial glucose and insulin levels (mean of postprandial iAUCs of breakfast, lunch, and dinner) under conditions of CR and OF (low vs. high ET for insulin: −28.9 and −44.5% both *p* < 0.001, and for glucose −34.2% (*p* < 0.001) and −29.7% (*p* = 0.066)). Changes in postprandial glucose and insulin response are shown in Fig. 4, separated for breakfast, lunch and dinner. After breakfast, postmeal glucose (*p* < 0.05) and insulin (*p* < 0.01) levels were lower with high compared with low ET during CR only, during OF only postprandial insulin response was lower with high compared with low ET (*p* < 0.05). After lunch, ET had no effect on postprandial glucose levels whereas postprandial insulin levels decreased with higher ET during all conditions of EB (all *p* < 0.05). After dinner, the response in postprandial glucose decreased with increasing ET under all conditions of EB (all *p* < 0.01) whereas a decrease in postprandial insulin levels with higher ET was only observed during OF (*p* < 0.001).

## Discussion

The main finding of the study was that higher ET improved postprandial glucose and insulin responses at all levels of EB (Fig. 4). The improvement of postprandial insulin levels was most pronounced after lunch and for glucose after dinner. Postprandial insulin sensitivity was therefore enhanced by low intensity physical activity especially after lunch and dinner. The higher GL at dinner might have contributed to a higher postprandial glycemia. It is therefore possible that physical activity-induced postprandial improvements in glycemia were most pronounced after dinner. The lack of a significant change in basal glycemia and HOMA suggests, that ET primarily improves glucose uptake in skeletal muscle. In addition, only healthy normoglycemic subjects were investigated which makes an intervention effect on basal glucose and insulin levels unlikely (Fig. 3).

### Timing and frequency of physical activity

Physical activity in our study was always performed in a postmeal situation. Colberg et al. described, that an increase in daily activity of low intensity improves blood glucose management and lowers postmeal hyperglycemia, especially when physical activity was performed directly after meals^38^. Because peak glucose values usually appear within 90 min postprandially^39^, the optimal timing for physical activity has been assumed to be 30 min after meal onset^40^. The benefit of a postmeal compared with a premeal bout of physical activity on glycemia might be due to the synergistic effect between insulin and NIMGU by muscular contraction^41^. Elevated blood glucose levels 2 h after lunch have been shown to increase the risk for cardiovascular events by 50% and the risk for mortality by 89% in patients with type 2 diabetes^42^. Because the exercise effect is insulin-independent, it works well in patients with diabetes, too^43–45^. Therefore postmeal exercise may be an effective way to improve glucose control in patients with type 2 diabetes^46^. Borror et al. recommended that individuals with type 2 diabetes should focus on increasing energy expenditure after the largest meal of the day. However, 15 min of postmeal walking (3 times per day) was more effective in lowering 3-h postdinner glucose levels than a 45-min walk in the morning or afternoon compared with control day in subjects at risk for impaired glucose tolerance (*p* < 0.01)^16^.

In the present study, postmeal glucose and insulin levels decreased most after lunch and dinner (Fig. 4). This effect could be due to a cumulative effect of walking intervals over the intervention day or it might be explained by the circadian difference insulin sensitivity. Healthy nondiabetic individuals have a higher insulin sensitivity in the morning and show a diurnal decrease in insulin sensitivity, which is in part the result of better β-cell responsiveness^11^. When exercise was performed after lunch, there was a minimal impact on glycemia over the following 24 h^47^. By contrast, exercise performed in the evening has been shown to reduce glycemia during exercise and the overnight period in patients with type 2 diabetes^13^. However, the glucose- and insulin-lowering effect of exercise after a meal does not seem to persist at the following meal suggesting that these improvements are short-lived^12^.

In summary, timing and frequency of physical activity seem to play a significant role for improvement of glucose metabolism. With respect to feasibility and preventive recommendations it is important that breaking up prolonged sitting with short bouts of walking or simply standing already has positive effects on glycemia^48–50^. The implementation of several smaller walking units is easier to realize in everyday life, especially in risk groups like older persons.

### Intensity and type of physical activity

In our study, a positive effect on postprandial glycemia was achieved even by low intensity walking on the treadmill at 4 km/h. Glucose uptake is enhanced with higher intensity of physical activity because of increased glucose delivery, transport, and metabolism^51^. Further studies are needed to clarify a dose–response relationship for light intensity activity thresholds^52^. The type of exercise involves different muscle groups and masses and may therefore differently affect the extend of the glucose lowering effect. In line with this presumption, climbing stairs led to a more rapid decrease in postprandial blood glucose levels compared with cycling^53^. In patients with type 2 diabetes^54^ as well as in healthy volunteers^9,10,55,56^, slow postmeal walking was able to lower postprandial glucose and insulin levels compared with sedentary controls. We found reduced postprandial insulin levels at high compared with low ET during CR and OF (Fig. 4d–f) although the intake of CHO was considerable higher (e.g., during OF at low ET 366 ± 52 g vs. 507 ± 85 g at high ET). This is likely due to improved NIMGU by physical activity.

### Physical activity and energy balance

So far, all studies that investigated the impact of low intensity physical activity on postprandial glycemia did not consider EB. Hence, the observed postmeal glycemia-lowering effect of physical activity could be also explained by a higher energy expenditure and a resulting caloric deficit. This negative energy balance alone could explain the improvement in glycemia. This assumption is supported by the results of Larsen et al. who observed the same effect on glycemia by 45 min of exercise after breakfast or an equal energy deficit achieved by a lower energy intake at breakfast^12^. Both conditions led to a similar decrease in postprandial glycemia after breakfast. However, in the present study CR (between −19.5 and −21.0% EB) did not improve postmeal levels of glucose or insulin compared with zero EB. Similarly, OF (between +24.2 and +27.6% EB) did not worsen postprandial glucose metabolism (Fig. 4). On the other hand, a positive EB can result from inactivity^57^ and thus might explain the deterioration in insulin sensitivity^22^.

Postprandial glucose (*p* = 0.066) and insulin levels (*p* < 0.001) were also improved by a high ET compared with a low ET during OF (Fig. 4). The positive effect of physical activity on postprandial glucose metabolism was therefore independent of EB. Similar to our findings, 25% OF combined with a single bout of exercise (~60 min of ergometer or treadmill at 60% of VO_2_ peak) also led to a 20% decrease in postprandial insulin AUC (*p* < 0.05), compared with OF alone^58^. Impaired glucose metabolism by OF is well established^59^ and may not have been detectable in our study because of the short duration and relatively low energy surplus.

### Strength and limitations of the study

A strength of our study was the randomized crossover study design that strictly controlled for possible confounding factors, such as EB, diet, exercise, and sleeping behavior. The use of healthy participants may limit the generalizability of the results to people with obesity or type 2 diabetes. Studies that did not control EB already provided some evidence that postprandial exercise is also effective to improve glucose control in patients with type 2 diabetes^43–46,54^.

Since our study only covers a 24-h intervention, the results are not transferable to long-term effects of physical activity. However, short-term changes in eating and motion behavior, are very common and realistic in everyday life^20,21^. In addition, the quite long walking duration on the treadmill during high ET (330 min) could appear unrealistic, but the stay in a metabolic chamber is an artificial setting that prevents other spontaneous physical activity of daily life.

## Conclusion

Low intensity postprandial physical activity is effective to lower postmeal glucose and insulin levels in healthy adults independent of EB. Since higher postprandial glycemia even below the diabetic threshold has been shown to be a risk factor for cardiovascular disease^1,2^, walking after the meals is an advisable preventive strategy.
