# Correction: Impact of energy turnover on the regulation of glucose homeostasis in healthy subjects

**DOI:** 10.1038/s41387-019-0094-9

**Published:** 2019-10-07

**Authors:** Franziska Büsing, Franziska Anna Hägele, Alessa Nas, Mario Hasler, Manfred James Müller, Anja Bosy-Westphal

**Affiliations:** 1 Institute of Human Nutrition and Food Science, Christian-Albrechts University of Kiel, Kiel, Germany; 2 Institute of Nutritional Medicine, University of Hohenheim, Stuttgart, Germany; 3 Applied Statistics, Faculty of Agricultural and Nutritional Sciences, Christian-Albrechts University of Kiel, Kiel, Germany

**Keywords:** Randomized controlled trials, Risk factors

**Correction to: Nutrition & Diabetes**

10.1038/s41387-019-0089-6 published online 08 August 2019

Since publication of this article the authors noted that the legend for Table 1 was incomplete, as the subtitle was missing. The complete table should appear as given below:

**Table 1:** Physical activity and daily energy-, macronutrient- and glycemic load intake during caloric restriction, zero energy balance and overfeeding with low, medium and high energy turnover (ET)

	Caloric restriction			Energy balance			Overfeeding		
	Low ET	Med ET	High ET	Low ET	Med ET	High ET	Low ET	Med ET	High ET
**Physical acticity**									
PAL	1.26 ± 0.06	1.52 ± 0.05	1.74 ± 0.07	1.30 ± 0.03	1.57 ± 0.04	1.75 ± 0.05	1.34 ± 0.05	1.55 ± 0.04	1.71 ± 0.05
Steps [counts/d]	545 ± 902	17 618 ± 557	34 527 ± 1847	464 ± 912	17 710 ± 219	34 414 ± 1539	436 ± 847	17 790 ± 382	34 821 ± 1568
**Diet**									
Energy intake [kcal/d]	1 792 ± 144	2 156 ± 78	2 494 ± 90	2 390 ± 97	2 863 ± 72	3 325 ± 177	2 986 ± 99	3 570 ± 183	4 146 ± 320
CHO intake [g/d]	219.4 ± 20.8	255.1 ± 37.3	308.2 ± 12.0	292.7 ± 13.9	350.1 ± 6.0	406.5 ± 20.3	366.2 ± 11.8	437.2 ± 22.1	506.7 ± 37.9
Fat intake [g/d]	70.5 ± 4.9	84.7 ± 2.6	97.7 ± 5.0	93.7 ± 3.3	112.8 ± 3.9	131.0 ± 7.5	117.3 ± 3.9	140.8 ± 7.0	163.8 ± 12.5
Protein intake [g/d]	66.1 ± 4.0	79.4 ± 3.3	92.0 ± 5.6	88.4 ± 0.0	106.1 ± 5.6	120.3 ± 9.3	109.8 ± 3.0	131.0 ± 8.6	153.3 ± 13.0
Glycemic load [g/d]	124.0 ± 8.9	149.2 ± 4.7	173.8 ± 7.0	166.0 ± 6.5	197.8 ± 5.7	228.9 ± 14.1	206.4 ± 7.0	247.4 ± 13.0	285.9 ± 24.0

Values are means ± SDs; *n* = 16; linear mixed model with multiple contrast tests, results of physical activity were compared at the same level of ET between all energy balance conditions (all *p* > 0.05) and within all energy balances conditions at three levels of ET (all significantly different at *p* < 0.001); results of dietary intake were compared at the same level of ET between all energy balance conditions (all significantly different at *p* < 0.001) and within all energy balances conditions at three levels of ET (all significantly different at *p* < 0.001); ET, energy turnover; CHO, carbohydrate

This has been corrected in both the PDF and HTML versions of the Article.

